# Comparative functional genomics of *Saccharomyces cerevisiae* reveals genetic determinants of stress tolerance and ethanol fermentation

**DOI:** 10.1128/spectrum.03632-25

**Published:** 2026-04-17

**Authors:** Ying Han, Xin-Qiu Zhou, Xing-Xing Tang, Meng-Jing Teng, Ping Xiang, Ruo-Tian Nie, Ya-Fei Pei, Ke Zhang, Dao-Qiong Zheng, Fan Yang

**Affiliations:** 1 Guizhou Key Laboratory of Microbial Resources Exploration in Fermentation Industry, Kweichow Moutai Group, Zunyi, China; 2 State Key Laboratory of Biobased Transportation Fuel Technology, Ocean College, Zhejiang University, Hangzhou, China; 3 State Key Laboratory of Biobased Transportation Fuel Technology, College of Life Science, Zhejiang University, Zhoushan, China; Agroscope, Nyon, Switzerland

**Keywords:** phenotypic diversity, genome sequencing, genomic evolution, horizontal gene transfer, genome duplication

## Abstract

*Saccharomyces cerevisiae* strains from diverse origins exhibit distinct phenotypic traits, providing valuable diversity and adaptability for industrial applications. Here, we conducted a comparative analysis of phenotypic and genomic features across 41 *S. cerevisiae* strains with clear industrial niche associations, aiming to identify genetic determinants underlying stress resistance and ethanol fermentation efficiency. These strains displayed niche-specific growth advantages under fermentation-related stress conditions, yet none showed broad tolerance. During ethanol fermentation using wheat and sorghum substrates, ethanol yields varied from 0.42 to 0.48 g ethanol/g glucose, with strains exhibiting superior maltose utilization achieving higher ethanol titers. Substantial variation was also observed in glycerol and acetic acid production, and a strong negative correlation was detected between their yields. Whole-genome sequencing revealed that chromosomal aberrations, DNA recombination-mediated chromosomal rearrangements, loss of heterozygosity, and gene gain or loss were major genetic factors contributing to phenotypic diversity. Furthermore, identification of novel genes acquired through horizontal gene transfer expanded the genetic repertoire of *Saccharomyces* strains. An additional *SOD2* gene obtained from *Torulaspora microellipsoides* contributed to oxidative stress tolerance. Furthermore, our results demonstrate that whole-genome duplication in *S. cerevisiae* enhances maltose utilization and ethanol production in starchy substrate fermentation. Together, these findings offer novel mechanistic insights into the genomic evolution of yeast in industrial/ecological niches.

**IMPORTANCE:**

This study systematically analyzed phenotypic diversity and genomic variations across 41 diverse *Saccharomyces cerevisiae* strains. Key findings include strain-specific stress resistance linked to ecological niches, a strong glycerol-acetic acid negative correlation in starchy substrate fermentation, horizontal transfer-acquired *SOD2* enhancing oxidative tolerance, and genome duplication boosting maltose utilization and ethanol yield. These results uncover niche-specific genetic mechanisms driving *S. cerevisiae* adaptive evolution and provide references for screening of strains with improved industrial traits.

## INTRODUCTION

The budding yeast *Saccharomyces cerevisiae* is extensively exploited in baking and alcoholic fermentation (encompassing Chinese Baijiu, beer, wine, and bioethanol production) and acts as a versatile chassis for the biosynthesis of high-value compounds (1–6). Diverse alcoholic fermentation processes impose distinct physiological demands on yeast strains: notably, Chinese Baijiu fermentation—the focus of this study—relies on starchy substrates (wheat and sorghum), thus requiring efficient maltose utilization and robust tolerance to fermentation-derived stressors (e.g., organic acids and ethanol) (7–10). In contrast, wine fermentation necessitates strains with the capacity to tolerate and ferment high concentrations of monosaccharides (glucose and fructose) abundant in grape must (11). Accumulating evidence has documented significant variations in *S. cerevisiae* with respect to tolerance to ethanol, hyperosmosis, heat, and low pH stress (2, 12, 13), and large-scale genomic surveys (involving hundreds to thousands of strains) have delineated the core genomic features underlying yeast genetic diversity (14–22). However, a critical knowledge gap persists: most large-scale studies lack targeted analyses of genotype-phenotype associations for industrial fermentation-relevant traits (e.g., maltose utilization on starchy substrates and tolerance to fermentation-associated stresses) and fail to establish a causal link between genomic variations and niche-specific adaptive evolution in major industrial fermentation systems.

To address this knowledge gap, we selected 41 *S. cerevisiae* strains with well-characterized industrial/ecological origins (including Baijiu, beer, wine, bioethanol, and sake fermentation, as well as natural/host-associated niches) for systematic investigation. We comprehensively assessed their stress resistance and ethanol fermentation performance in a wheat/sorghum-based medium that mimics the actual Chinese Baijiu production conditions. Furthermore, we quantified key fermentation parameters, including ethanol titer, glucose-to-ethanol conversion efficiency, and byproduct formation, and analyzed the correlations between stress tolerance and fermentation traits. Whole-genome sequencing was subsequently performed to elucidate the evolutionary relationships and genomic divergence among these strains, which enabled the identification of niche-specific genetic mechanisms driving the variations in stress resistance and ethanol fermentation performance of *S. cerevisiae* in starchy substrate-based fermentation systems.

## RESULTS

### Phenotypic variations of the selected 41 *S. cerevisiae* strains

As depicted in Table 1, the 41 *S. cerevisiae* strains used in this investigation were sourced from diverse geographic locations and industrial contexts (Table 1). We selected strains from all major industrial ethanol fermentation systems closely related to starchy substrate utilization, including Chinese Baijiu (SC1, F18, and F37), bioethanol (NY1308, YJS329, and NY1300), beer (F1, F8, and F39), sake (F5 and F31), bread (F23 and F34), and wine (F4 and F11) fermentation. Most of these strains are all widely used in industrial production or isolated from industrial fermentation substrates, with confirmed relevance to starchy biomass metabolism (e.g., maltose utilization) and fermentation stress tolerance—directly aligning with our study’s focus on industrial starchy substrate fermentation. We also included strains from natural (river water F49 and apple F55) and host-associated (oral cavity F58) niches as phylogenetic and ecological controls to compare and distinguish industrial niche-specific adaptive traits from basal ecological traits of *S. cerevisiae* (Table 1).

**TABLE 1:** *S. cerevisiae* strains used in this study

Strain	Strain bank number	Country/region	Source	Sporulation, %	Ploidy
F1	CGMCC 2.2	China	Beer yeast, top fermentation	40.4	Diploid
F4	CGMCC 2.69	Spain	Spanish Rolling wine yeast	5.8	Diploid
F5	CGMCC 2.126	China	Sake yeast	0	Diploid
F6	CGMCC 2.168	United Kingdom	Edible yeast	46.9	Diploid
F7	CGMCC 2.185	China	Fruit wine yeast	0	Diploid
F8	CGMCC 2.200	China	Top-fermenting beer yeast	0	Diploid
F9	CGMCC 2.345	China	Ergosterol production	59.8	Triploid
F10	CGMCC 2.394	Russia	Champagne yeast	0	Diploid
F11	CGMCC 2.411	China	Wine yeast	15.6	Diploid
F12	CGMCC 2.427	China	Alcohol using cane molasses	25.2	Diploid
F13	CGMCC 2.453	China	Awamori yeast	22.6	Triploid
F14	CGMCC 2.476	China	Dye factory	2.8	Diploid
F16	CGMCC 2.529	Poland	Alcohol using beet molasses	32.1	Triploid
F17	CGMCC 2.536	Russia	Alcohol using wood hydrolysate	33.7	Diploid
F18	CGMCC 2.54	China	Baijiu	0	Triploid
F19	CGMCC 2.548	Germany	Fermentation chemical factory	22.6	Diploid
F21	CGMCC 2.593	China	Alcohol using beet molasses	51.2	Diploid
F23	CGMCC 2.631	Germany	Bread	37.3	Triploid
F25	CGMCC 2.745	China	Liquor medicinal	55.9	Diploid
F26	CGMCC 2.773	China	White koji	71.6	Diploid
F29	CGMCC 2.1042	China	Feed yeast	31.3	Diploid
F30	CGMCC 2.119	China	Alcohol using cane molasses	93.3	Triploid
F31	CGMCC 2.1407	Japan	Sake	0	Diploid
F32	CGMCC 2.1416	United States	Red Star ADY	8.1	Diploid
F33	CGMCC 2.1417	Australia	Active dry yeast	69.6	Triploid
F34	CGMCC 2.1423	Japan	Bread yeast	0	Triploid
F35	CGMCC 2.1425	Japan	Lager yeast	0	Diploid
F36	CGMCC 2.1426	Japan	Whisky yeast	10.1	Triploid
F37	CGMCC 2.1427	Japan	Baijiu	98	Triploid
F38	CGMCC 2.1429	Japan	Alcohol yeast	64.3	Triploid
F39	CGMCC 2.145	China	Beer yeast	0	Diploid
F41	CGMCC 2.1527	China	Acid-resistant fruit wine yeast	10.1	Diploid
F44	CGMCC 2.1554	China	Inositol and pyridoxamine detection	0	Diploid
F46	CGMCC 2.1639	China	Huangjiu brewing yeast	9.7	Diploid
F49	CGMCC 2.3095	China	River water	66.7	Diploid
F55	CGMCC 2.3854	China	Apple	39.6	Tetraploid
F58	CGMCC 2.3973	China	Oral cavity	42..2	Diploid
SC1	This study	China	Maotai liquor mash	9	Diploid
YJS329	(23)	China	Bioethanol yeast	32.7	Diploid
NY1308	(21)	China	Bioethanol yeast	27.3	Diploid
NY1300	CICC 1300	China	Bioethanol yeast	49.6	Triploid

To determine whether strains from different origins display distinct robustness (defined as tolerance to fermentation-related and some other stressors), we first assessed the growth performance of 41 *S. cerevisiae* strains. Stressors were selected for their direct relevance to industrial ethanol fermentation: organic acids (acetic acid and lactic acid, major fermentation-derived inhibitors), ethanol (the target product), antifungal agents (bifonazole, nocodazole, miconazole; for reference), oxidative stressors (H₂O₂ and paraquat), furan derivatives (furfural and 5-hydroxymethyl-2-furfural, sugar hydrolysis inhibitors), elevated temperature (39°C) and low temperature (16°C) (temperature fluctuations are an inherent characteristic of the traditional Baijiu fermentation process), osmotic stress (0.7 M NaCl), heavy metals (6 mM CuSO₄ and 12 mM CdCl₂), and antibiotics (G418, hygromycin B; for reference). Tolerance to each inhibitor was quantified as relative biomass, defined as the ratio of biomass under stress to that under the control condition (YPD medium without inhibitor).

**Fig 1:**
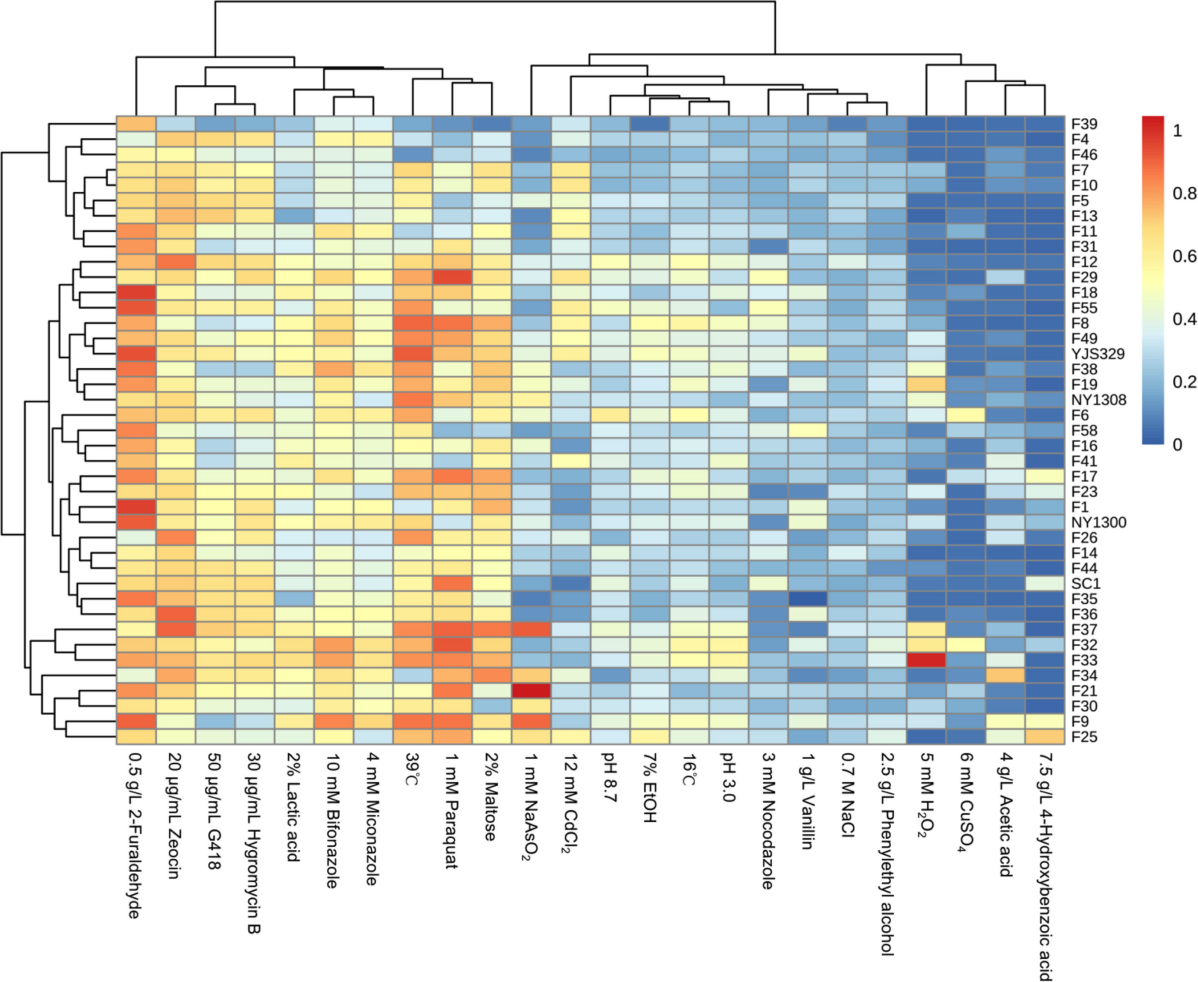
Comparison of stress resistance among 41 *S. cerevisiae* strains. Stress resistance was assessed by measuring the relative biomass formation of yeast strains grown under normal conditions (YPD medium) and various stress conditions. The heatmap was generated using an R script (pheatmap).

The results revealed that individual strains displayed niche-specific tolerance profiles, with specific strains showing superior resistance to particular fermentation-relevant inhibitors (Fig. 1). For example, strain F1 exhibited the highest tolerance to furfural but showed the lowest tolerance to vanillin, whereas strain F33 demonstrated greater resistance to H₂O₂ than other strains (Fig. 1). The strains with the highest resistance to ethanol, lactic acid, and acetic acid were F25, F33, and F34, respectively (Fig. 1). In contrast, the SC1 strain isolated from Moutai fermented grains did not display a competitive advantage under any tested stress conditions, suggesting that it lacks broad resistance to environmental stresses (Fig. 1). These results demonstrated that *S. cerevisiae* strains from different origins exhibit distinct strengths in fermentation-related stress tolerance, with no single strain showing high resistance to the 24 tested conditions.

Using clustering analysis, we identified consistent resistance behaviors to G418 and hygromycin B across all tested strains (Fig. 1). Similarly, resistance profiles were comparable between 4 g/L acetic acid and 7.5 g/L 4-hydroxybenzoic acid, as well as between 39°C and 1 mM paraquat, 16°C and pH 3, and 0.7 M NaCl and 2.5 g/L phenethyl alcohol. These findings suggest that yeast strains share common physiological mechanisms in tolerating these stressors. However, significant differences were observed in the resistance of these yeast strains to lactic acid and acetic acid (Fig. 1), both of which are common inhibitors in ethanol fermentation, indicating distinct toxic effects of these two organic acids on the yeast cells.

### *S. cerevisiae* strains showed different ethanol fermentation performance

**Fig 2:**
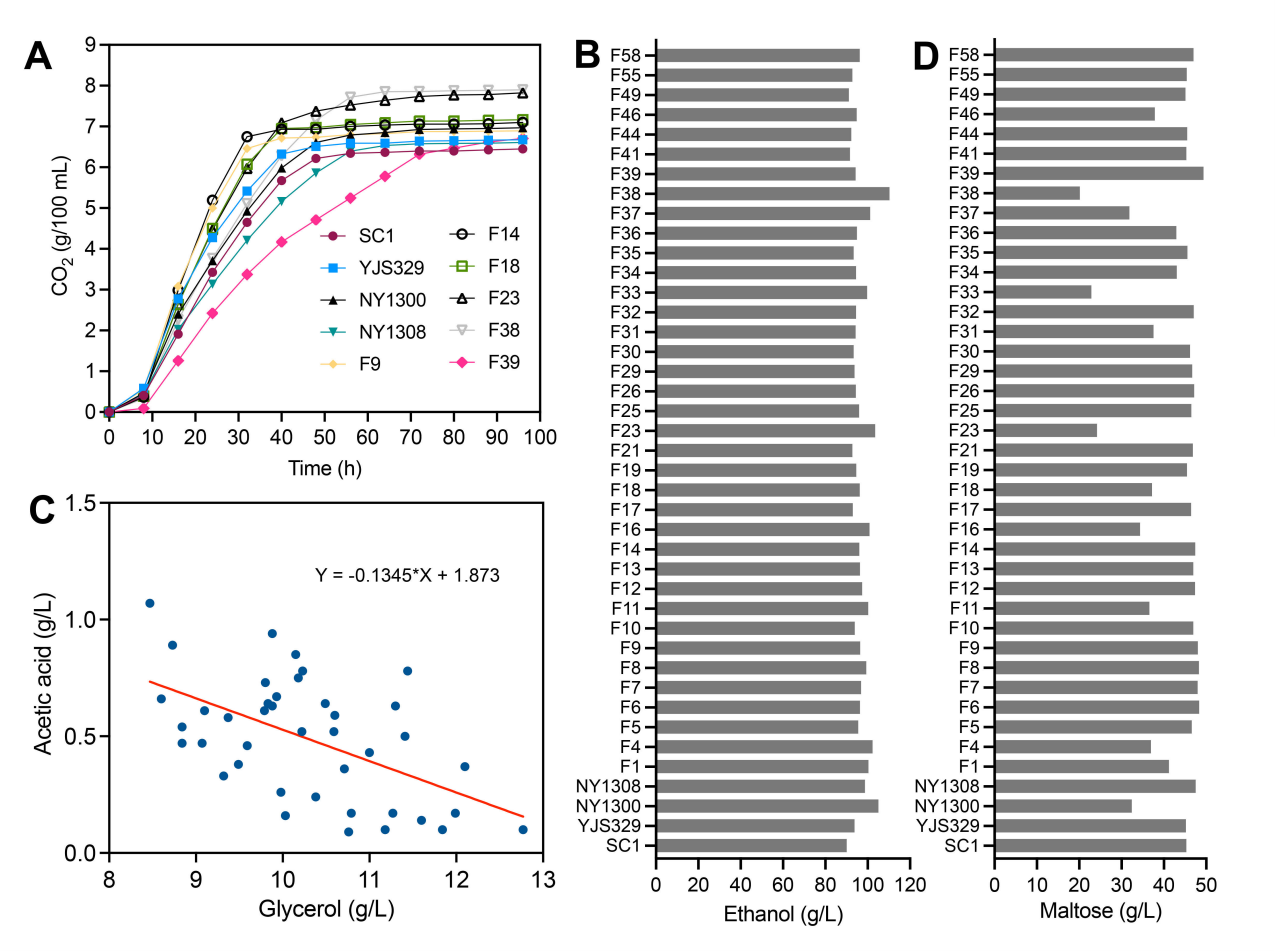
Ethanol fermentation performance of selected *S. cerevisiae* strains. (**A**) CO₂ loss (g/100 mL) during fermentation (0–96 h) of 10 representative strains. (**B**) Ethanol production (g/L) of 41 strains cultured in wheat or glutinous sorghum fermentation media. (**C**) Negative correlation between glycerol and acetic acid production. (**D**) Residual maltose concentration in the fermentation medium after 96 h of fermentation.

To compare the ethanol fermentation performance of the strains (mainly targeted to Baijiu production), we analyzed their fermentation rates and the production of ethanol and byproducts in a medium prepared by mixing wheat and sorghum in equal proportions (core raw materials for Baijiu), followed by enzymatic hydrolysis to obtain a mixture containing 180 g/L glucose and 65 g/L maltose. To illustrate the diverse ethanol fermentation kinetics among these strains, we selected 10 representative strains for depiction in Fig. 2A. Most strains completed ethanol fermentation within 56 h, ceasing CO_2_ production (Fig. 2A; Data set S1). However, strains like F14 exhibited faster fermentation rates than average, while others, such as F39, required more time to consume all glucose in the medium (Fig. 2A; Data set S1). The ethanol titer of these strains ranged from 90.1 g/L (SC1) to 110.3 g/L (F38) (Fig. 2B), corresponding to sugar-to-ethanol conversion efficiencies of 0.42–0.48 g ethanol/g glucose. Glycerol, the most abundantly produced byproduct, was detected at concentrations ranging from 8.6 g/L to 12.8 g/L (Data set S1), corresponding to conversion efficiencies of 0.05–0.1 g glycerol/g glucose. Acetic acid production also varied markedly among strains, with some producing as little as 0.14 g/L, while others accumulated up to 1.1 g/L (Data set S1). Correlation analysis revealed no significant association between ethanol titer and the titers of glycerol or acetic acid. However, a strong negative correlation was observed between glycerol and acetic acid (Fig. 2C). We also found that strains F1, F4, NY1300, and F38 exhibit significant maltose utilization capability (Fig. 2D). Among them, F38 converted the highest amount of maltose, achieving a final ethanol production of 110.3 g/L.

To examine the impact of lactic acid (a key inhibitor in Baijiu fermentation) on ethanol fermentation in these strains, 20 g/L lactic acid was added to the fermentation medium. In the fermentation process of aromatic Baijiu, more than 20 g/L of lactic acid can accumulate (24). We also attempted a concentration of 30 g/L, but found that none of the strains could initiate ethanol fermentation at this level. After the addition of 20 g/L lactic acid to the starchy substrate medium, most strains still completed glucose consumption within 96 h, while three strains (F1, F2, and F11) had residual glucose of 6–12 g/L due to their high sensitivity to lactic acid (Data set S2). In stress tests (Fig. 1), these strains also exhibited higher sensitivity to lactic acid, indicating a direct positive correlation between lactic acid tolerance and fermentation rate in its presence, a key finding for Baijiu fermentation strain screening.

### Association of stress tolerance and ethanol fermentation performance

**Fig 3:**
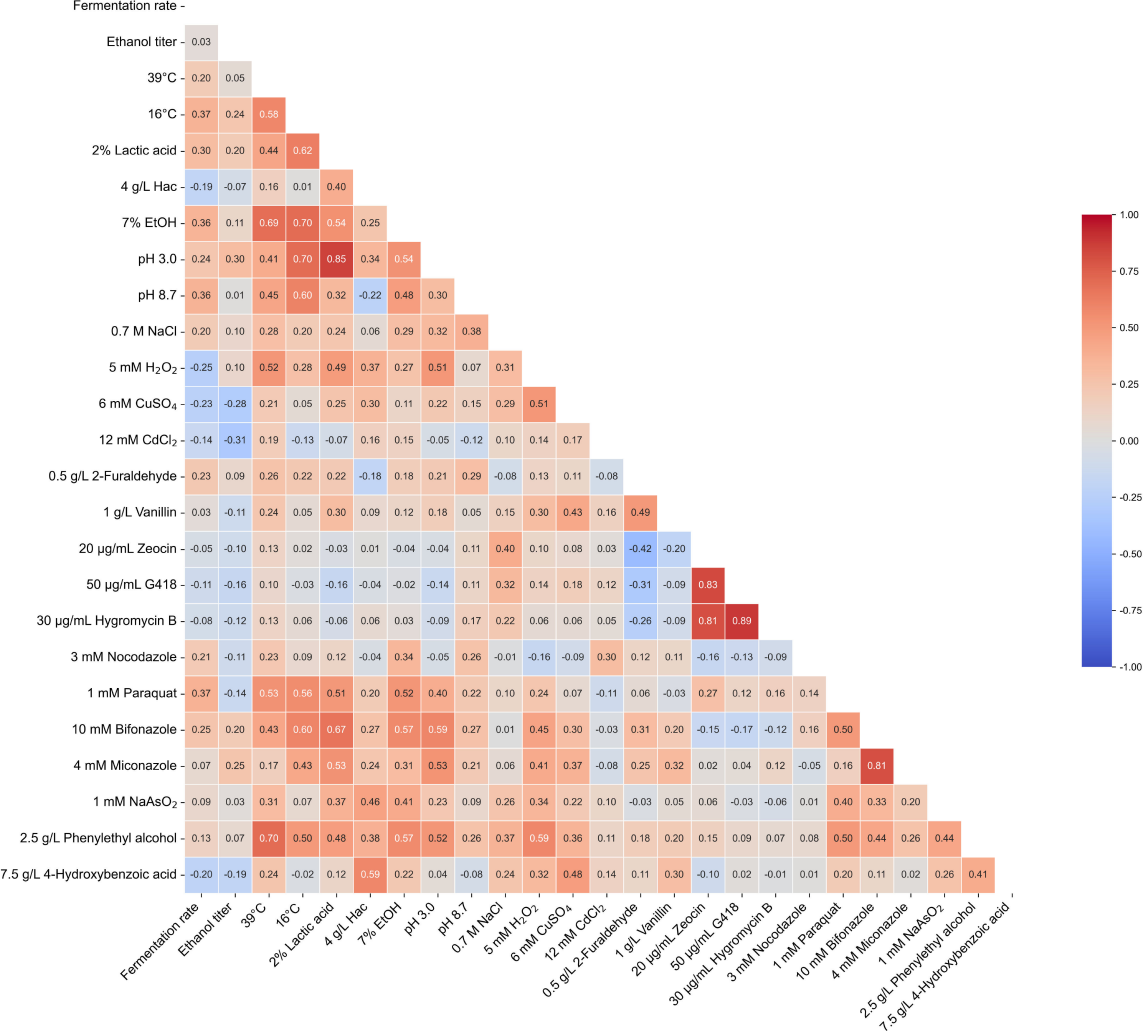
Correlation analysis of stress resistance and ethanol titer. Pearson correlation coefficients are displayed within the rectangles.

Whether resistance to fermentation-specific stressors of the selected strains correlates with ethanol fermentation performance in wheat/sorghum-based medium? Understanding this relationship is essential for developing effective screening strategies to identify *S. cerevisiae* strains with enhanced ethanol production capability. As shown in Fig. 3, correlation analysis revealed that the fermentation rate (defined as ethanol titer measured at the midpoint of the fermentation period) was significantly associated with resistance to 7% ethanol (*r* = 0.36), pH 8.7 (*r* = 0.36), 16°C (*r* = 0.37), and 1 mM paraquat (*r* = 0.37). In contrast, the final ethanol titer showed a stronger positive correlation with resistance to acidic conditions (pH 3, *r* = 0.30) than with resistance to 7% ethanol (*r* = 0.11) or other stressors. Under acidic conditions, yeast cells must expend substantial energy to pump out excess protons in order to mitigate low pH stress. Since energy production during anaerobic fermentation relies solely on glycolysis, this increased energy demand may explain the observed correlation between low pH and higher ethanol output. However, as the correlation is not particularly strong, our findings suggest that combinatorial stress conditions (a mix of two or more fermentation-relevant stressors), which mimic the complex stress environment of actual ethanol fermentation—may be a more effective strategy for screening high-ethanol-producing *S. cerevisiae* strains than single stressor screening.

### Whole-genome sequencing uncovers the genetic traits of the 41 *S. cerevisiae* strains

We performed high-throughput whole-genome sequencing on the *S. cerevisiae* strains listed in Table 1, with the primary goal of identifying genomic variations that underlie niche-specific stress tolerance and fermentation performance in starchy substrate systems. To identify genomic variations, the high-quality reads were analyzed using two complementary approaches: alignment to the reference genome S288C (https://www.yeastgenome.org) and *de novo* assembly. This enabled comprehensive detection of genomic alterations, including chromosomal aberrations, large-scale structural variations, loss of heterozygosity (LOH), single-nucleotide variations (SNVs), and small insertions and deletions (InDels).

#### Chromosomal aberrations and rearrangements

**Fig 4:**
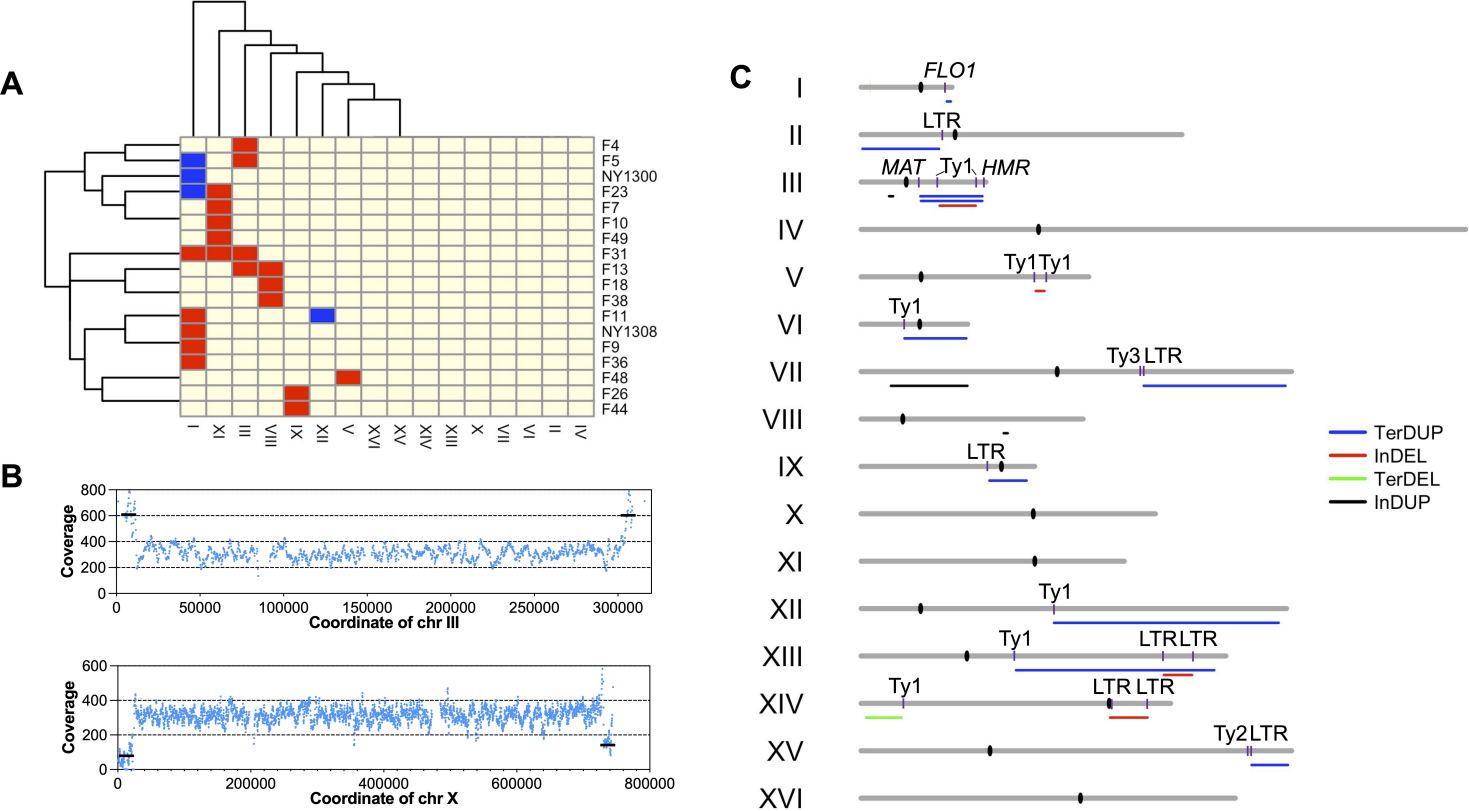
Genomic variations and evolutionary relationships among 41 *S. cerevisiae* strains. (**A**) Aneuploidy events were detected across the 41 strains. Red and blue indicate trisomy and monosomy events. (**B**) Representative examples of deletion and duplication events near telomeric regions. (**C**) Chromosomal rearrangements were identified among the 41 *S. cerevisiae* strains.

Using flow cytometry, we found that 12 out of the 41 *S. cerevisiae* strains were triploid, while strain F55 was identified as tetraploid (Table 1). In addition, by calculating sequencing read coverage, we identified 24 cases of whole-chromosome aneuploidy across the strain collection (Fig. 4A). For instance, in strain F5, we detected a case of monosomy, where one copy of a chromosome I was lost (Fig. 4A). Whole-chromosome aneuploidy resulted in copy number alterations affecting hundreds of genes in 18 out of 41 sequenced strains, highlighting its role as a major genetic factor influencing strain phenotypes. Among the 16 yeast chromosomes, chromosome III and chromosome XI were each amplified in four different strains. Chromosome I, the shortest in the yeast genome, showed alterations in eight different strains, making it the most frequently affected chromosome. These results suggested that the smaller ones (I, III, and VI) appear to be more prone to aneuploidy than the larger chromosomes, such as IV and VII.

Compared with the reference genome of strain S288C, each of the 41 strains exhibited copy number variations near the telomeric regions of several chromosomes, confirming the hyper-recombination activity of subtelomeric regions (25). For instance, in strain F41, the regions spanning 1–12 kb and 302–317 kb on chromosome III were duplicated, whereas the regions spanning 1–24 kb and 731–742 kb on chromosome X were deleted (Fig. 4B). In addition to the duplication and deletion events with breakpoints near telomeric regions, we also identified 18 rearrangement events unassociated with telomeres, including four interstitial deletions, three interstitial duplications, one terminal deletion, and ten terminal duplications (Fig. 4C; Data set S3). Sequence analysis of the regions flanking these breakpoints revealed that most were associated with repetitive DNA elements, such as long terminal repeats (LTRs), mating-type loci, and the transposon of yeast (Ty) elements (Fig. 4C). These findings suggest that unequal homologous recombination mediated by repetitive sequences is the primary genetic mechanism underlying the large-scale deletions and duplications in *S. cerevisiae* population.

**Fig 5:**
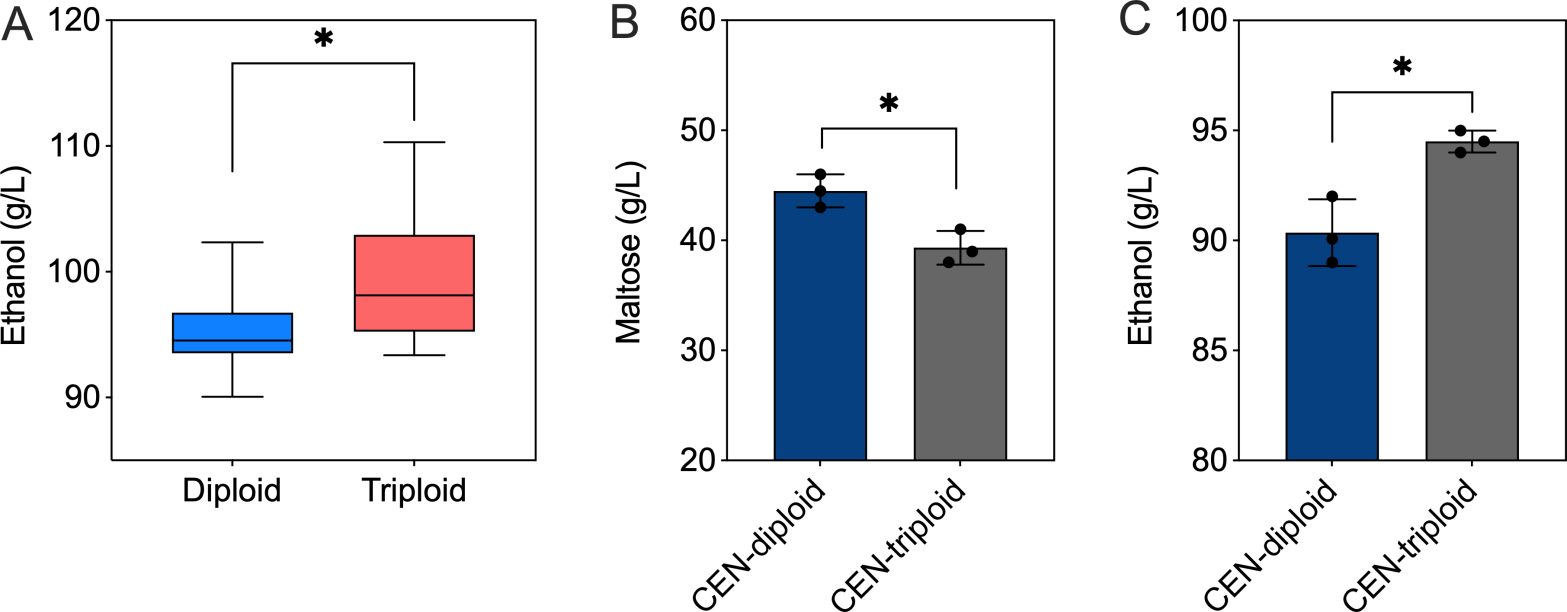
Genome duplication enhances maltose utilization and ethanol production. (**A**) Ethanol production of diploid and triploid *S. cerevisiae* strains. Data were obtained from Data set S1. (**B**) Residual maltose concentration in the fermentation medium after fermentation. CEN-diploid and CEN-triploid refer to *S. cerevisiae* strains with diploid and triploid genomes, respectively, constructed in our previous study. (**C**) Ethanol titers of CEN-diploid and CEN-triploid strains.

Considering the prevalence of ploidy variation and chromosomal aberrations in industrial *S. cerevisiae* strains (Table 1), we hypothesize that these genomic characteristics are regulators of ethanol fermentation performance in starchy substrate-based fermentation systems. In ethanol fermentation experiments, we found that maltose utilization during the late fermentation stage was a key determinant of the final ethanol yield (Fig. 2). Comparison of diploid and triploid strains revealed that triploids generally exhibited higher maltose consumption (Fig. 5A). In our previous study, we constructed homozygous diploid, triploid, and tetraploid strains from a haploid ancestor (26). Here, we specifically compared ethanol fermentation performance between diploid (CEN-diploid) and triploid (CEN-triploid) strains sharing the same genetic background. After 96 h of fermentation, the triploid strain consumed 11.6% more maltose than the diploid strain (Fig. 5B) and produced 4.5% more ethanol (Fig. 5C). These findings indicate that variation in ploidy among the 41 strains is an important factor influencing ethanol yield, particularly in media containing maltose as a major carbon source. However, we found that tetraploid yeast strains were much less frequent than diploid and triploid strains (Table 1), suggesting that further increases in ploidy do not confer an adaptive advantage. Our previous results also showed that tetraploid strains exhibit lower tolerance to stresses, such as ethanol, H_2_O_2_, acetic acid, and high temperature, compared to diploid and triploid strains (26). Moreover, other studies have reported that tetraploid strains display reduced genome stability, with higher frequencies of DNA mutations and chromosomal aberrations, which can compromise the stability of industrially relevant traits (27). These findings may help explain the lower prevalence of tetraploid strains compared to triploid strains.

#### SNVs and InDels

The 41 sequenced *S. cerevisiae* strains contained between 38,261 (F4) and 86,949 (NY1300) SNVs compared with the S288C reference genome (Data set S4). Strains YJS329 and F49 exhibited the closest evolutionary relationship, differing by only 3,671 SNVs. Assuming a generation time of 2 h and an SNV accumulation rate of approximately 5 × 10⁻³ per genome per cell division (28), the estimated divergence time among these strains ranges from approximately 168 to 3,970 years. Annotation of SNVs in each strain revealed that 63.9%–65.7% were located within coding regions (Data set S4). The ratio of synonymous to missense variants ranged from 1.67 to 1.95, indicating the latter are under stronger selection pressure. Among the 41 strains, 73–244 genes were affected by SNVs due to the gain/loss of start/end codon (Data set S5).

The number of InDels is significantly lower than that of SNVs, ranging from 1,446 to 3,415 per strain relative to the S288C reference genome (Data set S4). InDels were more frequently found in non-coding regions, accounting for approximately 82% of all cases (Data set S6). Notably, the vast majority of InDels occur at mononucleotide repeats [such as (A)_3-100_] or microsatellite loci [such as (AT)_3-100_], suggesting that they primarily result from replication slippage during DNA synthesis. Among these strains, 10%–14% InDels are located within coding regions; 63–194 InDels led to frameshift mutations, and 0–5 InDels resulted in start/end code gain/loss (Data set S6).

#### LOH is frequent among the *S. cerevisiae* strains

**Fig 6:**
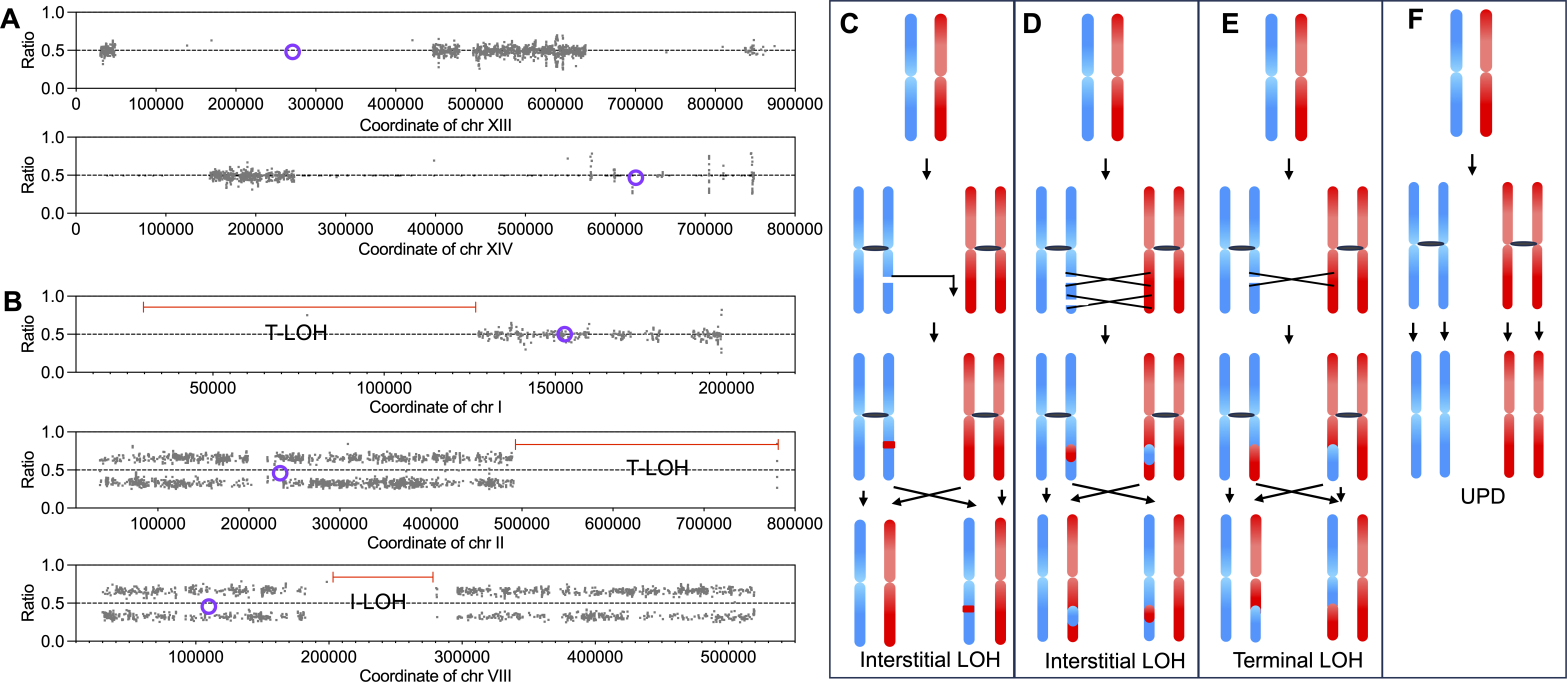
LOH events and genetic models. (**A**) Examples of LOH events likely induced by meiotic recombination in the strain F17. Violet circles represent centromeres. Gray points indicate heterozygous SNVs. (**B**) Examples of mitotic recombination-mediated LOH events in the strain F23. Red lines indicate LOH regions. (**C**) Genetic model of interstitial LOH resulting from gene conversion. (**D**) Double crossover led to interstitial LOH. (**E**) Genetic model of terminal LOH resulting from crossover. (**F**) Uniparental LOH events due to chromosome segregation errors.

*S. cerevisiae* exhibits two reproductive modes, namely sexual reproduction and asexual reproduction. Theoretically, both meiotic and mitotic DNA recombination can drive LOH in *S. cerevisiae*. However, the relative contributions of these two pathways to LOH in natural and laboratory populations remain incompletely characterized. Meiosis-associated LOH is more evenly distributed on chromosomes (examples from strain F17 were shown in Fig. 6A) (29), whereas mitotic LOH events occur far more frequently at telomeric regions than centromeric regions (28); these distinct distribution patterns allow us to identify the primary mechanism driving LOH in individual strains. In diploid yeast strains, an SNV site with approximately 50% read support suggests a heterozygous site, whereas in triploid strains, heterozygous sites may exhibit read support levels around 33% or 66%. To evaluate the homozygosity of these genomes, we applied a threshold of 90% SNVs read support: regions with support below this threshold were classified as heterozygous, while those equal to or above 90% were considered homozygous. We found that the proportion of homozygous regions in the genomes of these yeast strains ranged from 13% to 100% (Data set S7; the heterozygous regions of each chromosome of strains are listed here), indicating that LOH events have occurred throughout their evolutionary history. In strains F6, F23, F36, F38, and SC1 (low sporulation efficiency; Table 1), the centromeric regions tended to remain heterozygous, whereas near telomeric regions were largely homozygous (terminal LOH) (Data set S7), suggesting that mitotic recombination played a major role in shaping LOH patterns. In contrast, strains F11, F17, F29, and F44 (moderate/high sporulation efficiency; Table 1) exhibited a more uniform distribution of LOH regions across the genome, indicating that meiotic recombination was likely the predominant mechanism driving LOH in this strain. Several strains, such as F26, F30, and YJS329 (very high sporulation efficiency, >70%; Table 1), exhibit genomes that are almost entirely homozygous, providing further evidence for the role of meiosis in shaping their genomic evolution.

LOH events occurring within internal chromosomal regions, typically spanning only a few kilobases, are defined as interstitial LOH (I-LOH) (Fig. 6B). In contrast, crossover-mediated LOH events, which yield homozygosity extending to chromosomal termini, are designated as terminal LOH (T-LOH) (Fig. 6B). These two LOH subtypes are driven by distinct homologous recombination mechanisms: I-LOH is primarily mediated by gene conversion (Fig. 6C) and double crossover (Fig. 6D), while T-LOH arises from chromosomal crossover (Fig. 6E). Notably, in several strains, we observed a high level of heterozygosity across most chromosomes, with only one or a small number of chromosomes displaying marked homozygosity (e.g., chromosome XVI in strains F36 and NY1308, and chromosomes III, V, and IX in strain F7). This phenomenon may result from a uniparental disomy (UPD) event (Fig. 6F), in which both sister chromatids of a chromosome are mistakenly segregated into the same daughter cell during mitosis. In wild-type diploid cells, the frequency of reciprocal UPD for chromosome V has been estimated to be approximately 10⁻^7^ per cell division (30). Alternatively, UPD may arise through an initial chromosome loss event, followed by chromosome duplication during subsequent cell divisions. These results confirm the contribution of UPD to large-scale LOH in *S. cerevisiae* strains. Such events may accelerate adaptive evolution by converting heterozygous loci to homozygosity, thereby fixing beneficial alleles under selective pressure in specific niches.

#### Identification of additional genes among *S. cerevisiae* strains

**Fig 7:**
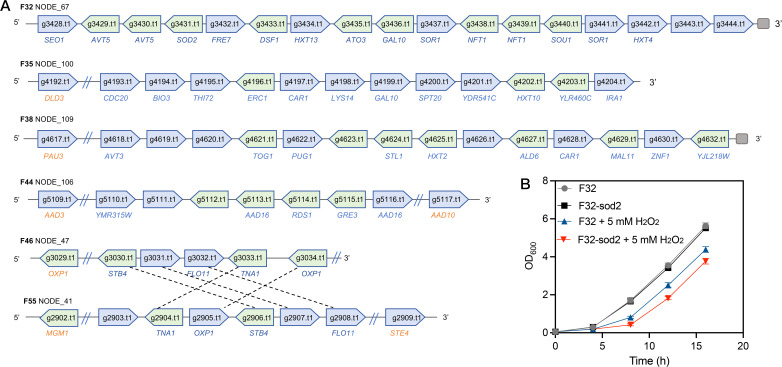
Additional genes absent from the S288C genome. (**A**) Examples of additional genes located in the contigs NODE_67, NODE_100, NODE_109, NODE_106, NODE_47, and NODE_41 in strains F32, F35, F38, F44, F46, and F55. Gray rectangles represent telomeres. Gene names highlighted in orange show high (>85%) identity to those on the S288C genome (i.e., these are not additional genes). Gene highlighted in blue shows low (≤85%) or no identity to those on the S288C genome. (**B**) Growth of F32 and *sod2* mutants in 25 mL YPD with or without 5 mM H_2_O_2_. The initial OD_600_ is 0.05.

To compare strain-specific gene pool differences among selected strains, which may potentially underlie their divergent stress tolerance phenotypes, we conducted *de novo* genome assembly and open reading frame (ORF) prediction for 41 *S. cerevisiae* isolates. Data set S8 presents the genome sizes (11.1 to 11.34 Mb) and numbers of ORFs (5,363 to 5,581) for these strains. Compared to the reference genome S288c, the sequenced 41 *S. cerevisiae* strains had 1,810 additional genes (≤85% sequence identity to the ORFs in S288C), with each strain’s genome containing 18–97 additional genes (Data set S8). In strain F32, we identified a 61-kb DNA fragment (contig number is NODE_67) absent from the S288C reference genome (Fig. 7A). The leftmost 77 bp of this contig could be aligned to three repeated regions of S288C genome (921,339–921,415 bp on chromosome XIII, 1,081,527–1,081,603 bp on chromosome XV, and 9,619–9,543 bp on chromosome XVI), whereas the rightmost region consisted of telomeric repeats, indicating this contig was located at chromosome end in this strain. Annotation using Augustus software predicted that this fragment encodes 17 genes, including *SEO1*, *AVT5*, *SOD2*, *FRE7*, *DSF1*, *HXT13*, *ATO3*, *GAL10*, *SOR1*, *NFT1*, *SOU1*, and *HXT4* (Fig. 7A). *SOU1* was first reported to encode a sorbose reductase required for the utilization of L-sorbose in yeasts, such as *Candida albicans* (29). This enzyme catalyzes the conversion of L-sorbose to D-sorbitol using NADPH as a cofactor and plays a key role in the metabolic pathway that enables yeasts to use L-sorbose as a carbon source. Adjacent to *SOU1*, we also identified a gene (*SOR1*) encoding sorbitol dehydrogenase (Fig. 7A), which catalyzes the oxidation of D-sorbitol to fructose. These findings suggest that multiple genes located on contig NODE_67 (sequence is provided in Data set S9) may collectively confer the ability of strain F32 to utilize L-sorbose. DNA sequence similarity analysis revealed high homology of this contig with *Torulaspora microellipsoides*, suggesting that the fragment likely originated through HGT. Although more than 3,000 *S. cerevisiae* strains have been sequenced and assembled to date (5), this fragment has not been detected in any other strain. We also discovered similar horizontally transferred fragments in other strains, as shown in Fig. 7A. These results suggest that such yeast strains have undergone interspecies hybridization events during their evolutionary history. In strain F46, we identified a horizontally transferred fragment flanked on both sides by the *OXP1* gene, indicating that HGT not only introduces exogenous genes but may also lead to copy number variation of native genes (Fig. 7A). In strain F55, contig NODE_41 contains five genes identical to those found in contig NODE_41 of strain F46, but arranged in a different order on the chromosome. This observation can be explained by the possibility that these five genes existed as a circular DNA molecule prior to chromosomal integration and were later inserted into the same chromosomal region during evolution. This phenomenon was first reported in the study by Borneman et al. (14).

The additional genes illustrated in Fig. 6A were annotated into several groups, including membrane transporters (*HXT2*, *HXT4*, *HXT13*, *AVT3*, *AVT5*, *PUG1*, *THI72*, *ERC1*, and *STL1*), enzymes involved in carbon source utilization (*BGL1*, *SOU1*, and *SOR1*), superoxide dismutase (*SOD2*), transcription factors (*RDS1*, *LYS14*, *STB4*, and *TOG1*), and others. It is likely that these genes may expand the spectrum of carbon sources utilized by yeast and enhance stress tolerance, thereby increasing adaptability to diverse environments. Although it was not feasible to experimentally validate the effects of all horizontally transferred genes, we specifically deleted the additional *SOD2* gene from *T. microellipsoides* acquired in strain F32 to examine its phenotypic impact. The deletion of this horizontally transferred *SOD2* gene did not affect cell growth under normal conditions; however, in the presence of 5 mM H₂O₂, the mutant exhibited slower growth and less biomass formation than strain F32 (Fig. 7B). These results indicate that the acquired *SOD2* gene contributes to the high resistance of strain F32 to oxidative stress (Fig. 1). In summary, functional validation of the additional genes across yeast populations will deepen our understanding of how horizontal gene transfer contributes to phenotypic evolution in *S. cerevisiae*.

## DISCUSSION

Through comparative analyses of 41 *S. cerevisiae* strains from diverse backgrounds, this study addressed the knowledge gap of genotype-phenotype associations for fermentation-relevant traits in wheat/sorghum-based systems. These strains not only harbor numerous point mutations (SNVs and InDels) but also exhibit varying degrees of aneuploidy, large-scale DNA copy number variations, and strain-specific additional genes acquired via HGT. Certain genomic alterations—including the genome duplication and the acquisition of novel genes through HGT—were identified as niche-specific genetic determinants of enhanced ethanol titer and stress tolerance in starchy substrate fermentation. Below, we contextualize these findings and discuss their implications for both basic science and biotechnological applications.

### Phenotypic diversity: a reflection of ecological adaptation and industrial relevance

Our results showed the absence of a “super strain” with broad tolerance to all 24 tested stressors. This pattern aligns with the “trade-off” hypothesis of microbial adaptation—yeast prioritize fitness in their native environments over generalist tolerance (31). For example, strains isolated from Baijiu fermentation (e.g., SC1 and F18) showed poor sporulation and no standout stress resistance, likely because the continuous, complex solid-state fermentation environment of Baijiu selects for stable, non-sporulating phenotypes rather than broad stress tolerance. In contrast, bioethanol strains (NY1308 and YJS329) displayed enhanced ethanol tolerance, reflecting selection for traits critical to their industrial niches.

In ethanol fermentation, the 0.42–0.48 g ethanol/g glucose conversion efficiency range is consistent with industrial benchmarks, but the marked variation in byproduct production—particularly the strong negative correlation between glycerol and acetic acid yields—highlights a key metabolic trade-off. Glycerol synthesis consumes NADH to maintain redox balance during anaerobic fermentation, while acetic acid production is linked to acetyl-CoA shunting or pyruvate decarboxylase activity (32, 33). The inverse relationship between these metabolites suggests that strains fine-tune NADH allocation to balance redox homeostasis and byproduct accumulation, a trait with direct industrial relevance: low acetic acid (to avoid yeast inhibition) and moderate glycerol (to limit carbon loss) are ideal for high ethanol yields. Additionally, strains with superior maltose utilization (F38 and NY1300) achieved higher ethanol titers, emphasizing maltose as a rate-limiting carbon source in wheat-sorghum-based fermentation (e.g., Chinese Baijiu). This finding is particularly valuable for industries relying on starchy substrates, where efficient hydrolysis of maltose directly determines process efficiency.

### Genomic variations: drivers of phenotypic plasticity among *S. cerevisiae* strains

Through whole-genome sequencing, we identified four major genetic mechanisms that likely drive fermentation-relevant phenotypic diversity in starchy substrate systems: ploidy/chromosomal aberrations, SNVs/InDels, LOH, and additional gene gain. Collectively, these genetic adaptations explain the rapid evolutionary plasticity of *S. cerevisiae* in response to the distinct stress pressures and metabolic demands imposed by industrial fermentation environments.

Aneuploidy was widespread among the 41 strains, with 12 triploids, 1 tetraploid (F55), and 23 cases of whole-chromosome copy number changes. Notably, the smallest chromosome (I) was the most frequently aneuploid, while larger chromosomes (IV and VII) were rarely altered. This pattern supports the hypothesis that small chromosomes, which carry fewer essential genes, are more tolerant to copy number changes (26, 34). Previous studies have shown that certain aneuploidy events enhance environmental adaptability (34–36). For example, monosomy of chromosome IX confers resistance to 5-HMF (37), while trisomy of chromosome III is associated with increased ethanol tolerance (38, 39). It is likely that the observed amplification of chromosome III in strains F4, F5, F13, and F31 (Fig. 4A) may reflect selection of these strains under high-ethanol conditions. We also identified 17 non-telomeric rearrangements linked to repetitive elements, indicating that unequal homologous recombination drives large-scale genomic plasticity. Notably, we found that triploid strains consistently outperformed diploids in maltose utilization (11.6% higher consumption) and ethanol production (4.5% higher titer), while tetraploids were rare and exhibited poor stress tolerance. This aligns with our previous work showing that genome duplication modulates gene expression and stress responses (30) and extends it to a key industrial trait for starchy substrate fermentation: maltose metabolism. The enhanced maltose utilization in triploids likely stems from increased dosage of *MAL* family genes, which are rate-limiting for maltose catabolism (19). In contrast, tetraploids suffer from genomic instability—including higher mutation rates and chromosomal aberrations (40)—which compromises the consistency of industrial traits (e.g., ethanol yield, stress tolerance). This “ploidy sweet spot” (triploid > diploid > tetraploid) has direct implications for strain breeding: generating triploid strains via crosses or genome duplication could be a simple, effective strategy to improve performance in starchy-substrate fermentations.

LOH emerged as another critical driver of fermentation niche adaptation, with strains exhibiting 13%–100% genome homozygosity and a direct link between sporulation efficiency. Meiotic recombination dominated LOH in high-sporulation strains, leading to genome-wide homozygosity, while mitotic recombination caused telomere-proximal LOH in low-sporulation strains. This distinction highlights LOH as a context-dependent strategy: meiotic LOH accelerates adaptation in sexual populations by fixing beneficial alleles, while mitotic LOH provides asexual strains with a mechanism to purge deleterious mutations or amplify advantageous ones. UPD events in certain strains further expand LOH diversity by generating whole-chromosome homozygosity that may accelerate the fixation of beneficial alleles for fermentation-relevant stress tolerance.

Previous studies have identified point mutations as a critical genetic mechanism modulating stress tolerance in yeast. For instance, via whole-genome sequencing of laboratory-evolved *S. cerevisiae* strains, Salas-Navarrete et al. revealed that mutations in the 3′ untranslated regions (3′ UTRs) of *RAS2* and *HSF1* confer resistance to heat and acid stress (17). In the present study, we did not perform targeted manipulation of specific point mutations to validate their functional impacts on yeast stress tolerance and ethanol fermentation. Nevertheless, our genomic analyses indicated that a considerable number of SNVs and InDels likely act as a key factor perturbing gene function—through inducing frameshift mutations and disrupting start/stop codons—and such variations affect up to 7% of all protein-coding genes in individual strains. The fixation of these mutations during adaptive evolution may therefore represent an effective evolutionary strategy for yeast to enhance cellular robustness and optimize ethanol fermentation performance.

HGT emerged as a major contributor to phenotypic innovation, with 1,810 additional genes (compared to the S288C genome) identified across the 41 strains. The most striking example is the acquisition of *SOD2* from *T. microellipsoides* in strain F32, which directly enhanced oxidative stress tolerance. This finding extends previous work by Milner et al. (24), who showed that HGT of transporter genes expands yeast carbon source utilization, by demonstrating that HGT also boosts stress resistance—a trait critical for industrial fermentations where reactive oxygen species accumulate due to high temperature or inhibitor exposure. Another notable HGT event in F32 involves *SOU1* and *SOR1*, which together enable L-sorbose utilization. While L-sorbose is not a major component of wheat-sorghum substrates, this pathway may confer an advantage in natural environments or specialized fermentations where polyols are abundant.

### Conclusion

Our work demonstrates that *S. cerevisiae*’s phenotypic diversity for fermentation-relevant traits in starchy substrate systems arises from a combination of multiple types of genetic events. By bridging genotype and phenotype for key industrial traits (maltose utilization and fermentation-related stress tolerance), this study fills a critical knowledge gap in yeast comparative genomics and provides targeted, actionable strategies for engineering strains that meet the demands of starchy substrate-based industrial ethanol fermentation.

## MATERIALS AND METHODS

### Strains and medium

Most of the yeast strains listed in Table 1 were obtained from the China General Microbiological Culture Collection Center (CGMCC). Strains NY1308 and YJS329 are two bioethanol strains isolated in our previous studies (16, 23). The yeast strains were cultured in YPD medium consisting of 20 g/L glucose, 20 g/L peptone, and 10 g/L yeast extract.

To assess stress tolerance, yeast cells were cultivated in 25 mL of liquid YPD medium with or without specific stressors at an initial OD₆₀₀ of 0.05. Biomass formation was monitored by measuring the OD₆₀₀ using a spectrophotometer. For ethanol fermentation, wheat and glutinous sorghum were mixed at a 1:1 mass ratio and homogenized with double-distilled water at a 2:1 (wt/vol) ratio. The mixture was soaked at room temperature for 30 min. Liquefying enzyme (6 U/g substrate; 20,000 U/mL) was added, and the slurry was heated with continuous stirring to 80°C for pre-gelatinization. The mixture was then cooked at 105°C for 1 h to complete gelatinization. After cooling to 95°C, an equivalent dose of liquefying enzyme was added again. Upon cooling to 60°C, saccharifying enzyme (200 U/g substrate; 200,000 U/mL) was introduced, and saccharification was carried out at 55°C for 3 h. The hydrolysate was filtered through a sieve, and the resulting filtrate was collected as the fermentation medium.

### Ethanol fermentation and metabolite analysis

*S. cerevisiae* cells were precultured in 15 mL of YPD medium for 20 h. The cells were harvested by centrifugation (4,000 × *g*, 5 min) and inoculated into 120 mL of fermentation medium at an initial OD₆₀₀ of 0.2. Fermentation was carried out at 30°C with shaking at 120 rpm. The weight of each flask was measured every 8 h to monitor fermentation progress via CO₂ loss. Samples (5 mL) of fermentation broth were collected, centrifuged at 12,000 rpm for 5 min, and the supernatant was filtered through 0.45-μm membranes. Ethanol, lactic acid, glucose, glycerol, and acetic acid concentrations were determined by HPLC using a Bio-Rad Aminex HPX-87H column (300 mm × 7.8 mm) maintained at 50°C. The mobile phase consisted of 4 mM H₂SO₄ at a flow rate of 0.6 mL/min, and analytes were detected using a refractive index detector.

### Flow cytometry analysis

Flow cytometry was performed to determine the ploidy of *S. cerevisiae* strains. Yeast cells were cultured overnight in YPD medium at 30°C with shaking at 200 rpm. Cells (1 mL) were collected by centrifugation (4,000 × *g*, 5 min). The cell pellets were resuspended in 70% (vol/vol) ethanol and fixed at 4°C overnight. Fixed cells were washed twice with 50 mM sodium citrate buffer (pH 7.4) and resuspended in the same buffer containing 0.25 mg/mL RNase A (Thermo Fisher Scientific). The suspension was incubated at 37°C for 2 h to degrade RNA. Subsequently, propidium iodide (PI; 50 µg/mL final concentration) was added to stain DNA, and the samples were incubated in the dark at room temperature for 30 min. Flow cytometric analysis was performed using a BD Accuri C6 flow cytometer (BD Biosciences, USA) with excitation at 488 nm and emission collected at 585 ± 20 nm. Data were analyzed using FlowJo software (v10.8). The fluorescence intensity histograms were used to estimate relative DNA content, with haploid (*1n*) and diploid (*2n*) control strains serving as references to determine the ploidy level of each strain.

### Whole-genome sequencing

*S. cerevisiae* cells cultured overnight in 5 mL of YPD medium were used for genomic DNA extraction with the Genomic DNA Extraction Kit (Omega Bio-Tek, Norcross, GA, USA). DNA quality and concentration were assessed by agarose gel electrophoresis. Indexed sequencing libraries were prepared by ligating adapters from the MGI Adapter Set 8 (Vazyme, Nanjing, China). Library construction was performed on the MGISP-960 automated platform (MGI, Shenzhen, China) using the VAHTS Universal Plus DNA Library Prep Kit for MGI (Vazyme, Nanjing, China). Library quality and concentration were verified using the Qubit dsDNA HS Assay Kit (Thermo Fisher Scientific, MA, USA). Circularized libraries were generated with the VAHTS Circularization Kit for MGI (Vazyme, Nanjing, China), and DNA nanoballs (DNBs) were prepared using the MGISEQ-2000RS High Throughput Sequencing Kit (MGI, Shenzhen, China). Sequencing was performed on the MGISEQ-2000 platform (MGI, Shenzhen, China) using a paired-end 2 × 150 bp strategy. Raw reads were quality-checked with FastQC (v0.11.9) and trimmed with Trimmomatic (v0.39) to remove adapters and bases with Phred quality scores below 20.

### SNVs and InDels calling

High-quality reads were aligned to the *S. cerevisiae* reference genome using the BWA-MEM algorithm with default parameters (41). The resulting SAM files were converted to BAM format and sorted using SAMtools (42). SNVs and InDels were identified using VarScan (43). Variant annotation was performed with SnpEff (44), utilizing a pre-annotated S288C genome database to predict the potential effects of each variant on coding sequences and regulatory regions.

### Genome assembly and ORF prediction

The genomes of the yeast strains were *de novo* assembled using SPAdes (45). ORFs were predicted with Augustus (46) and functionally annotated by mapping them to the non-redundant protein database using BLAST (47).

### Gene deletion

The deletion cassette of the *SOD2* gene was constructed by one-step PCR using the primers 5′-ATGTCTGTCGACCGTATTTCTTCGGTGGTTAAAAAAGATAGCTCTTCAACTGCAGGTCGACAACCCTT-3′ and 5′-TCAATAATTGAGCAATTTGGCGCCATCAAACTTTTTAGACGCCTCAGCCGTGGATCTGATATCACCT-3′. The plasmid pUG6, which carries the *KanMX6* selection marker, was used as the template (48). The resulting PCR product was introduced into *S. cerevisiae* cells using the lithium acetate/single-stranded DNA/polyethylene glycol transformation method (49). Transformants were selected on solid YPD medium supplemented with 300 mg/L G418. Correct integration of the deletion cassette was verified by diagnostic PCR and confirmed through DNA sequencing.

## Data Availability

The raw data of whole genome sequencing of *S. cerevisiae* isolates were deposited in the SRA database with the accession number PRJNA1345352.

## SUPPLEMENTAL MATERIAL

Supplemental material spectrum.03632-25-s0001.xlsx Data sets S1 to S9.

ASM does not own the copyrights to Supplemental Material that may be linked to, or accessed through, an article. The authors have granted ASM a non-exclusive, world-wide license to publish the Supplemental Material files. Please contact the corresponding author directly for reuse.

## References

[1] Wohlbach DJ, Rovinskiy N, Lewis JA, Sardi M, Schackwitz WS, Martin JA, Deshpande S, Daum CG, Lipzen A, Sato TK, Gasch AP. 2014. Comparative genomics of Saccharomyces cerevisiae natural isolates for bioenergy production. Genome Biol Evol 6:2557–2566. doi:10.1093/gbe/evu199 25364804 PMC4202335

[2] Ibáñez C, Pérez-Torrado R, Chiva R, Guillamón JM, Barrio E, Querol A. 2014. Comparative genomic analysis of Saccharomyces cerevisiae yeasts isolated from fermentations of traditional beverages unveils different adaptive strategies. Int J Food Microbiol 171:129–135. doi:10.1016/j.ijfoodmicro.2013.10.023 24334254

[3] Marsit S, Leducq JB, Durand É, Marchant A, Filteau M, Landry CR. 2017. Evolutionary biology through the lens of budding yeast comparative genomics. Nat Rev Genet 18:581–598. doi:10.1038/nrg.2017.49 28714481

[4] Morard M, Pérez-Través L, Perpiñá C, Lairón-Peris M, Collado MC, Pérez-Torrado R, Querol A. 2023. Comparative genomics of infective Saccharomyces cerevisiae strains reveals their food origin. Sci Rep 13:10435. doi:10.1038/s41598-023-36857-z 37369738 PMC10300040

[5] Loegler V, Friedrich A, Schacherer J. 2024. Overview of the Saccharomyces cerevisiae population structure through the lens of 3,034 genomes. G3 (Bethesda) 14:jkae245. doi:10.1093/g3journal/jkae245 39559979 PMC11631439

[6] Heasley LR, Argueso JL. 2022. Bursts of genomic instability potentiate phenotypic and genomic diversification in Saccharomyces cerevisiae. Front Genet 13:912851. doi:10.3389/fgene.2022.912851 35783258 PMC9247159

[7] Magalhães F, Vidgren V, Ruohonen L, Gibson B. 2016. Maltose and maltotriose utilisation by group I strains of the hybrid lager yeast Saccharomyces pastorianus. FEMS Yeast Res 16:fow053. doi:10.1093/femsyr/fow053 27364826 PMC5815069

[8] Zhu X, Chen L, Yang P, Luo S, Teng M, Zhu W, Li Y, Zhao D, Wang N, Chen X, Cheng M, Tu H, Huang W, Yang F, Wang L, Liu X, Ning K. 2025. Microbiome catalog and dynamics of the Chinese liquor fermentation process. Bioresour Technol 431:132620. doi:10.1016/j.biortech.2025.132620 40334798

[9] Yang F, Chen L, Liu Y, Li J, Wang L, Chen J. 2019. Identification of microorganisms producing lactic acid during solid-state fermentation of Maotai flavour liquor. J Inst Brew 125:171–177. doi:10.1002/jib.537

[10] Zhou Y, Hua J. 2025. Regulation and mechanisms of L-lactic acid and D-lactic acid production in Baijiu brewing: insights for flavor optimization and industrial application. Fermentation 11:213. doi:10.3390/fermentation11040213

[11] Tofalo R, Chaves-López C, Di Fabio F, Schirone M, Felis GE, Torriani S, Paparella A, Suzzi G. 2009. Molecular identification and osmotolerant profile of wine yeasts that ferment a high sugar grape must. Int J Food Microbiol 130:179–187. doi:10.1016/j.ijfoodmicro.2009.01.024 19230999

[12] Zheng DQ, Wu XC, Tao XL, Wang PM, Li P, Chi XQ, Li YD, Yan QF, Zhao YH. 2011. Screening and construction of Saccharomyces cerevisiae strains with improved multi-tolerance and bioethanol fermentation performance. Bioresour Technol 102:3020–3027. doi:10.1016/j.biortech.2010.09.122 20980141

[13] McIlwain SJ, Peris D, Sardi M, Moskvin OV, Zhan F, Myers KS, Riley NM, Buzzell A, Parreiras LS, Ong IM, Landick R, Coon JJ, Gasch AP, Sato TK, Hittinger CT. 2016. Genome sequence and analysis of a stress-tolerant, wild-derived strain of Saccharomyces cerevisiae used in biofuels research. G3 (Bethesda) 6:1757–1766. doi:10.1534/g3.116.029389 27172212 PMC4889671

[14] Borneman AR, Desany BA, Riches D, Affourtit JP, Forgan AH, Pretorius IS, Egholm M, Chambers PJ. 2011. Whole-genome comparison reveals novel genetic elements that characterize the genome of industrial strains of Saccharomyces cerevisiae. PLoS Genet 7:e1001287. doi:10.1371/journal.pgen.1001287 21304888 PMC3033381

[15] Peter J, De Chiara M, Friedrich A, Yue J-X, Pflieger D, Bergström A, Sigwalt A, Barre B, Freel K, Llored A, Cruaud C, Labadie K, Aury J-M, Istace B, Lebrigand K, Barbry P, Engelen S, Lemainque A, Wincker P, Liti G, Schacherer J. 2018. Genome evolution across 1,011 Saccharomyces cerevisiae isolates. Nature 556:339–344. doi:10.1038/s41586-018-0030-5 29643504 PMC6784862

[16] Gallone B, Steensels J, Prahl T, Soriaga L, Saels V, Herrera-Malaver B, Merlevede A, Roncoroni M, Voordeckers K, Miraglia L, Teiling C, Steffy B, Taylor M, Schwartz A, Richardson T, White C, Baele G, Maere S, Verstrepen KJ. 2016. Domestication and divergence of Saccharomyces cerevisiae beer yeasts. Cell 166:1397–1410. doi:10.1016/j.cell.2016.08.020 27610566 PMC5018251

[17] Otero JM, Vongsangnak W, Asadollahi MA, Olivares-Hernandes R, Maury J, Farinelli L, Barlocher L, Osterås M, Schalk M, Clark A, Nielsen J. 2010. Whole genome sequencing of Saccharomyces cerevisiae: from genotype to phenotype for improved metabolic engineering applications. BMC Genomics 11:723. doi:10.1186/1471-2164-11-723 21176163 PMC3022925

[18] Higgins P, Grace CA, Lee SA, Goddard MR. 2021. Whole-genome sequencing from the New Zealand Saccharomyces cerevisiae population reveals the genomic impacts of novel microbial range expansion. G3 Genes Genomes Genet 11:jkaa027. doi:10.1093/g3journal/jkaa027 PMC7849907 33561237

[19] Babrzadeh F, Jalili R, Wang C, Shokralla S, Pierce S, Robinson-Mosher A, Nyren P, Shafer RW, Basso LC, de Amorim HV, de Oliveira AJ, Davis RW, Ronaghi M, Gharizadeh B, Stambuk BU. 2012. Whole-genome sequencing of the efficient industrial fuel-ethanol fermentative Saccharomyces cerevisiae strain CAT-1. Mol Genet Genomics 287:485–494. doi:10.1007/s00438-012-0695-7 22562254

[20] Heasley LR, Argueso JL. 2022. Genomic characterization of a wild diploid isolate of Saccharomyces cerevisiae reveals an extensive and dynamic landscape of structural variation. Genetics 220:iyab193. doi:10.1093/genetics/iyab193 34791219 PMC9176296

[21] Zhang K, Di YN, Qi L, Sui Y, Wang TY, Fan L, Lv ZM, Wu XC, Wang PM, Zheng DQ. 2018. Genetic characterization and modification of a bioethanol-producing Saccharomyces cerevisiae strain. Appl Microbiol Biotechnol 102:2213–2223. doi:10.1007/s00253-017-8727-1 29333587

[22] Salas-Navarrete PC, de Oca Miranda AIM, Martínez A, Caspeta L. 2022. Evolutionary and reverse engineering to increase Saccharomyces cerevisiae tolerance to acetic acid, acidic pH, and high temperature. Appl Microbiol Biotechnol 106:383–399. doi:10.1007/s00253-021-11730-z 34913993

[23] Zheng DQ, Wang PM, Chen J, Zhang K, Liu TZ, Wu XC, Li YD, Zhao YH. 2012. Genome sequencing and genetic breeding of a bioethanol Saccharomyces cerevisiae strain YJS329. BMC Genomics 13:1–13. doi:10.1186/1471-2164-13-479 22978491 PMC3484046

[24] Milner DS, Attah V, Cook E, Maguire F, Savory FR, Morrison M, Müller CA, Foster PG, Talbot NJ, Leonard G, Richards TA. 2019. Environment-dependent fitness gains can be driven by horizontal gene transfer of transporter-encoding genes. Proc Natl Acad Sci USA 116:5613–5622. doi:10.1073/pnas.1815994116 30842288 PMC6431176

[25] Argueso JL, Carazzolle MF, Mieczkowski PA, Duarte FM, Netto OVC, Missawa SK, Galzerani F, Costa GGL, Vidal RO, Noronha MF, Dominska M, Andrietta MGS, Andrietta SR, Cunha AF, Gomes LH, Tavares FCA, Alcarde AR, Dietrich FS, McCusker JH, Petes TD, Pereira GAG. 2009. Genome structure of a Saccharomyces cerevisiae strain widely used in bioethanol production. Genome Res 19:2258–2270. doi:10.1101/gr.091777.109 19812109 PMC2792172

[26] Zhang K, Fang YH, Gao KH, Sui Y, Zheng DQ, Wu XC. 2017. Effects of genome duplication on phenotypes and industrial applications of Saccharomyces cerevisiae strains. Appl Microbiol Biotechnol 101:5405–5414. doi:10.1007/s00253-017-8284-7 28429058

[27] Hirota S, Nakayama Y, Ekino K, Harashima S. 2024. Highly genomic instability of super-polyploid strains of Saccharomyces cerevisiae. J Biosci Bioeng 137:77–84. doi:10.1016/j.jbiosc.2023.11.009 38135639

[28] Sui Y, Qi L, Wu J-K, Wen X-P, Tang X-X, Ma Z-J, Wu X-C, Zhang K, Kokoska RJ, Zheng D-Q, Petes TD. 2020. Genome-wide mapping of spontaneous genetic alterations in diploid Saccharomyces cerevisiae cells. Proc Natl Acad Sci USA 117:28191–28200. doi:10.1073/pnas.2018633117 33106417 PMC7668089

[29] Zhang K, Wu XC, Zheng DQ, Petes TD. 2017. Effects of temperature on the meiotic recombination landscape of the yeast Saccharomyces cerevisiae. mBio 8:e02099-17. doi:10.1128/mBio.02099-17 29259092 PMC5736917

[30] Andersen SL, Petes TD. 2012. Reciprocal uniparental disomy in yeast. Proc Natl Acad Sci USA 109:9947–9952. doi:10.1073/pnas.1207736109 22665764 PMC3382553

[31] Abram F, Arcari T, Guerreiro D, O’Byrne CP. 2021. Evolutionary trade-offs between growth and survival: The delicate balance between reproductive success and longevity in bacteria. Adv Microb Physiol 79:133–162. doi:10.1016/bs.ampbs.2021.07.002 34836610

[32] Vemuri GN, Eiteman MA, McEwen JE, Olsson L, Nielsen J. 2007. Increasing NADH oxidation reduces overflow metabolism in Saccharomyces cerevisiae. Proc Natl Acad Sci USA 104:2402–2407. doi:10.1073/pnas.0607469104 17287356 PMC1892921

[33] Geertman J-MA, van Dijken JP, Pronk JT. 2006. Engineering NADH metabolism in Saccharomyces cerevisiae: formate as an electron donor for glycerol production by anaerobic, glucose-limited chemostat cultures. FEMS Yeast Res 6:1193–1203. doi:10.1111/j.1567-1364.2006.00124.x 17156016

[34] Gilchrist C, Stelkens R. 2019. Aneuploidy in yeast: segregation error or adaptation mechanism? Yeast 36:525–539. doi:10.1002/yea.3427 31199875 PMC6772139

[35] Tsai HJ, Nelliat A. 2019. A double-edged sword: aneuploidy is a prevalent strategy in fungal adaptation. Genes (Basel) 10:787. doi:10.3390/genes10100787 31658789 PMC6826469

[36] Berman J. 2016. Ploidy plasticity: a rapid and reversible strategy for adaptation to stress. FEMS Yeast Res 16:fow020. doi:10.1093/femsyr/fow020 26945893

[37] Zhu YX, He M, Li KJ, Wang YK, Qian N, Wang ZF, Sheng H, Sui Y, Zhang DD, Zhang K, Qi L, Zheng DQ. 2024. Novel insights into the effects of 5-hydroxymethylfurfural on genomic instability and phenotypic evolution using a Saccharomyces cerevisiae model. Appl Environ Microbiol 90:e0164923. doi:10.1128/aem.01649-23 38108644 PMC10807415

[38] Albillos-Arenal S, Alonso Del Real J, Adam AC, Barrio E, Querol A. 2025. Chromosome III aneuploidy enhances ethanol tolerance in industrial Saccharomyces cerevisiae by increasing TUP1 expression. bioRxiv:2025. 10.1111/1751-7915.70244 PMC12514340 41074565

[39] Morard M, Macías LG, Adam AC, Lairón-Peris M, Pérez-Torrado R, Toft C, Barrio E. 2019. Aneuploidy and ethanol tolerance in Saccharomyces cerevisiae. Front Genet 10:82. doi:10.3389/fgene.2019.00082 30809248 PMC6379819

[40] Janbon G, Sherman F, Rustchenko E. 1998. Monosomy of a specific chromosome determines L-sorbose utilization: a novel regulatory mechanism in Candida albicans. Proc Natl Acad Sci USA 95:5150–5155. doi:10.1073/pnas.95.9.5150 9560244 PMC20229

[41] Li H, Durbin R. 2009. Fast and accurate short read alignment with Burrows-Wheeler transform. Bioinformatics 25:1754–1760. doi:10.1093/bioinformatics/btp324 19451168 PMC2705234

[42] Li H, Handsaker B, Wysoker A, Fennell T, Ruan J, Homer N, Marth G, Abecasis G, Durbin R, 1000 Genome Project Data Processing Subgroup. 2009. The sequence Alignment/Map format and SAMtools. Bioinformatics 25:2078–2079. doi:10.1093/bioinformatics/btp352 19505943 PMC2723002

[43] Koboldt DC, Zhang Q, Larson DE, Shen D, McLellan MD, Lin L, Miller CA, Mardis ER, Ding L, Wilson RK. 2012. VarScan 2: somatic mutation and copy number alteration discovery in cancer by exome sequencing. Genome Res 22:568–576. doi:10.1101/gr.129684.111 22300766 PMC3290792

[44] Cingolani P, Platts A, Wang LL, Coon M, Nguyen T, Wang L, Land SJ, Lu X, Ruden DM. 2012. A program for annotating and predicting the effects of single nucleotide polymorphisms, SnpEff: SNPs in the genome of Drosophila melanogaster strain w1118; iso-2; iso-3. Fly (Austin) 6:80–92. doi:10.4161/fly.19695 22728672 PMC3679285

[45] Bankevich A, Nurk S, Antipov D, Gurevich AA, Dvorkin M, Kulikov AS, Lesin VM, Nikolenko SI, Pham S, Prjibelski AD, Pyshkin AV, Sirotkin AV, Vyahhi N, Tesler G, Alekseyev MA, Pevzner PA. 2012. SPAdes: a new genome assembly algorithm and its applications to single-cell sequencing. J Comput Biol 19:455–477. doi:10.1089/cmb.2012.0021 22506599 PMC3342519

[46] Stanke M, Steinkamp R, Waack S, Morgenstern B. 2004. AUGUSTUS: a web server for gene finding in eukaryotes. Nucleic Acids Res 32:W309–12. doi:10.1093/nar/gkh379 15215400 PMC441517

[47] Ye J, McGinnis S, Madden TL. 2006. BLAST: improvements for better sequence analysis. Nucleic Acids Res 34:W6–W9. doi:10.1093/nar/gkl164 16845079 PMC1538791

[48] Gueldener U, Heinisch J, Koehler GJ, Voss D, Hegemann JH. 2002. A second set of loxP marker cassettes for Cre-mediated multiple gene knockouts in budding yeast. Nucleic Acids Res 30:e23. doi:10.1093/nar/30.6.e23 11884642 PMC101367

[49] Gietz RD, Schiestl RH. 2007. High-efficiency yeast transformation using the LiAc/SS carrier DNA/PEG method. Nat Protoc 2:31–34. doi:10.1038/nprot.2007.13 17401334

